# Sevoflurane prolonged the QTc interval and increased transmural dispersion of repolarization in a patient with long QT syndrome 3: a case report

**DOI:** 10.1186/s40981-017-0093-6

**Published:** 2017-05-18

**Authors:** Atsuhiro Kitaura, Shinichi Nakao, Shinichi Hamasaki, Kei Houri, Takatoshi Tsujimoto, Seishi Kimura, Mayuka Matsushima

**Affiliations:** Department of Anesthesiology, Kindai University Faculty of Medicine, 377-2 Ono-Higashi, Osaka-Sayama, 589-8511 Osaka Japan

**Keywords:** Long QT syndrome type 3, Torsades de pointes, Sevoflurane, QTc, Tp-e, Arrhythmia

## Abstract

We report that sevoflurane not only caused marked QTc interval prolongation but also increased transmural dispersion of repolarization in a patient with long QT syndrome 3 (LQT3). A 16-year-old male with LQT3 underwent a shoulder operation. He experienced no episode of syncope or cardiac arrest, but his preoperative electrocardiography (ECG) showed marked QTc interval prolongation (631 ms) and Tp-e interval prolongation (126 ms). Anesthesia was induced with propofol and maintained with 2% sevoflurane and remifentanil. Although no lethal arrhythmias occurred in the perioperative period, not only the QTc interval but also Tp-e interval was further prolonged by sevoflurane. While sevoflurane has been recognized as a safe anesthetic in terms of QT interval prolongation, even in patients with long QT syndromes, we believe that sevoflurane might be avoided for poorly controlled LQT3 patients.

## Background

Congenital long QT syndrome (LQTS) is a genetic disease characterized by a prolonged QT interval on electrocardiography (ECG), lethal arrhythmias, such as torsades de pointes (TdP) and ventricular fibrillation (VF), and higher chance of sudden cardiac death [1]. To date, at least 15 different genes have been reported to be associated with LQTS [1]. The most frequent LQTS subtypes are type 1 (LQT1), which accounts for 42% of all LQTS cases, type 2 (LQT2), which accounts for 45%, and type 3 (LQT3), which accounts for 5% [2]. Unlike LQT1 and LQT2, which are caused by potassium channel mutations, mutation of the cardiac sodium channel SCN5A has been shown to underlie LQT3 pathogenesis [1]. In LQT3, symptoms such as syncope and cardiac arrest are most frequently observed during rest or at night, and bradycardia is a risk factor for TdP and VF [1, 2]. However, to the best of our knowledge, there have been few reports on the anesthetic management of LQT3 cases.

We describe the anesthetic management of a patient with LQT3, and in addition report the effects of sevoflurane, which inhibits delayed rectifier potassium channels [3, 4], on both the QTc interval and the interval between the peak and end of the T wave (Tp-e), a surrogate of ventricular transmural dispersion of repolarization [5, 6].

## Case presentation

**Fig. 1:**
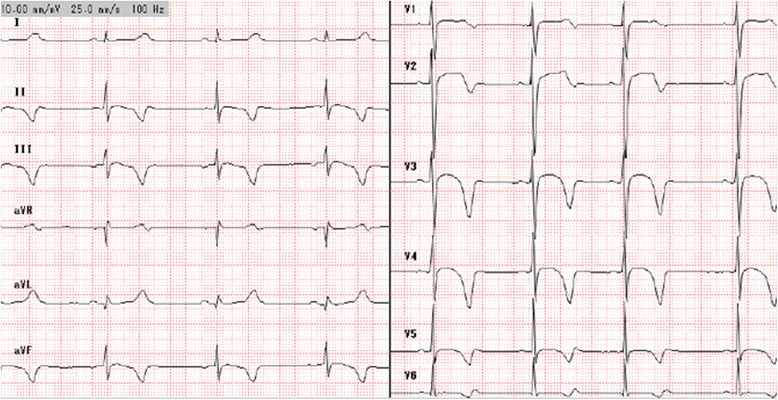
12-lead ECG performed before the operation. The ECG shows not only the obvious QTc interval prolongation (QTc 631 ms by Bazett formula) but also prolongation of the Tp-e interval (Tp-e 126 ms)

A 16-year-old male (height, 166 cm; weight, 60 kg), who had been diagnosed with congenital long QT syndrome type3 (LQT 3) by ECG and genetic examination, was scheduled to undergo the removal of the implant in his right shoulder and had consulted a doctor in the pediatrics department of our hospital. He had not experienced any episode of syncope or cardiac arrest, and he had not been administered any medication due to the refusal of both himself and his mother. His 12-lead ECG performed before the operation showed not only the obvious QTc interval prolongation (QTc 631 ms by Bazett formula) but also prolongation of the Tp-e interval (Tp-e 126 ms) (Fig. 1). Other than abnormal ECG, his laboratory studies including his serum electrolytes level and physical examinations were within the normal limits.

In the operating room, standard monitors were used, including BP cuff, pulse oximetry, and ECG (II lead). Anesthesia was induced with 100 mg propofol and 0.5 μg/kg/min remifentanil, tracheal intubation was performed after administration of 50 mg rocuronium, and anesthesia was maintained with 2% sevoflurane in 40% O_2_ and approximately 0.3 μg/kg/min remifentanil. End-tidal CO_2_ (ETCO_2_) was maintained between 35 and 40 mmHg.

**Fig. 2:**
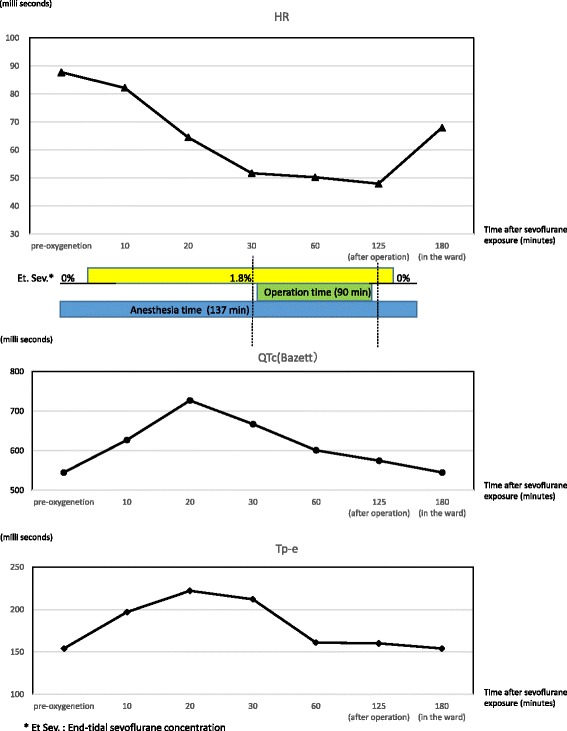
Time courses of the QTc interval, the Tp-e interval, and the HR interval during anesthesia. The QTc interval, the Tp-e interval was also prolonged after sevoflurane exposure, with peaks of QTc (727 ms) and Tp-e (222 ms) at 20 min after sevoflurane exposure. Both the QTc and the Tp-e intervals gradually decreased with time and returned to around the pre-anesthetic levels 40 min after sevoflurane administration

RR interval, QT interval, which is from the onset of the QRS complex to the end of the T wave, and Tp-e interval, which is from the peak to the end of the T wave, were measured manually. The QT interval was adjusted for the patient’s heart rate using Bazett’s formula (QTc = QT/[RR/1,000]^1/2^). The time courses of the QTc interval, the Tp-e interval, and the HR interval during anesthesia are shown in Fig. 2. In addition to the QTc interval, the Tp-e interval was also prolonged after sevoflurane exposure, with peaks of QTc (727 ms) and Tp-e (222 ms) at 20 min after sevoflurane exposure. However, we did not dare to increase his HR or use mexiletine or lidocaine during the anesthesia, because his HR was more than his ordinary HR, and both the QTc and the Tp-e intervals gradually decreased with time and returned to around the pre-anesthetic levels 40 min after sevoflurane administration. There was no remarkable change of his serum electrolyte level throughout the operation (e.g., Na 140 mEq/L, K 3.6 mEq/L, Cl 104 mEq/L, Ca 9.8 mEq/L). The anesthesia time was 2 h and 17 min. The operation time was one and a half hours. The bleeding volume was 50 g. The patient’s intra-operative and post-operative courses were uneventful.

### Discussion

In the present case, we report that in addition to the QTc interval, the Tp-e interval was prolonged by sevoflurane in a poorly controlled patient with LQT3.

The management and treatment of patients with LQT3 differs from those with LQT1 and LQT2 due to the differences in pathophysiology. Consequently, anesthetic management of patients with LQT3 for preventing lethal arrhythmias is also quite different. Unlike LQT1 and LQT2, bradycardia is a risk factor of TdP in LQT3 cases, and β-blockers are not very effective in preventing lethal arrhythmias [1, 2]. Instead, several studies have shown that mexiletine or flecainide, Na^+^ channel blockers, significantly shorten the QTc interval [7, 8]. Furthermore, it has been demonstrated that patients with LQT3 are more likely to experience lethal arrhythmias than patients with LQT1 and LQT2, and the use of implantable cardioverter-defibrillators (ICDs) is recommended [9, 10]. On the other hand, the present patient was not administered with the above drugs, because he had not experienced any episode of syncope or lethal arrhythmias, such as TdP or VF, and his mother refused such treatment. However, his QTc interval and Tp-e interval prolongations were so marked that the doctor in charge tried to persuade the patient to take the drugs, but failed.

Recent studies have demonstrated that susceptibility to TdP arises from the induction of afterdepolarization and increased dispersion of ventricular repolarization, rather than QT interval prolongation per se [11]*.* Tp-e interval prolongation is now recognized as a good indicator of the risk of TdP, because the Tp-e interval is a surrogate of transmural dispersion of repolarization (TDR) across the myocardial wall [12]. While neither the normal range of Tp-e interval nor the indications for the need of correction by heart rate have been defined yet, mean Tp-e intervals in healthy children or adults have been reported to be approximately 65 or 76 ms, respectively [13–15]. Therefore, the Tp-e interval of the present patient, 126 ms, was markedly prolonged even before anesthesia and was further prolonged to a critical level to induce lethal arrhythmias by sevoflurane.

Sevoflurane inhibits HERG (human *ether-a-go-go-related gene*) currents (I_Kr_), LQT1/minK currents (I_Ks_), and Kv4.3 currents (Ito) [3, 4], and induces significant QT interval prolongation. However, as the prolongation is modest and sevoflurane does not affect the Tp-e interval in healthy people [5, 6], sevoflurane is generally considered as a safe general anesthetic in terms of TdP and/or lethal arrhythmias, even in those with LQTS [16]. This is probably the first report which clearly demonstrates the Tp-e interval prolongation by sevoflurane not only in healthy people but also in patients with LQTS. We think of two reasons why the Tp-e interval is prolonged by sevoflurane: The case is a patient with LQTS 3, which is considered to be more dangerous than patients with LQT 1 and LQT 2, or the patient is poorly controlled preoperatively in terms of the QTc and Tp-e intervals. Because LQTS is relatively uncommon (1 in 2500), there is a lack of a robust evidence based on appropriate perioperative management, and there are few reports which systemically investigate the effect of sevoflurane on the QTc and the Tp-e intervals in LQTS patients. Whyte et al. reported that general anesthesia for LQTS patients was performed 128 of 158 (81%) with volatile anesthetics including sevoflurane without any serious arrhythmias, such as TdP [17]. However, TdP has been reported to be induced by sevoflurane in some patients, including in a patient with LQT2 [18], a patient with LQTS who received fluconazole infusion [19], and a patient with poorly controled diabetes [20], because both fluconazole and hyperglycemia inhibit potassium channels (Ikr channels) and induce QT interval prolongation [21, 22]. Furthermore, Kang et al. demonstrated that sevoflurane and DPI 201-106, which reduces Na^+^ channel inactivation and mimicks LQT3, prolong action potential duration, and the combination of the two drugs has been shown to have an effect on guinea-pig hearts greater than an additive effect of their activities, indicating the potential risk for ventricular arrhythmias when sevoflurane is used for LQT3 patients [23]. On the other hand, because propofol at clinically relevant concentrations neither affects Ikr [3] nor prolongs QTc interval [5, 6], propofol might have been used in the present patient, rather than sevoflurane.

## Conclusions

Sevoflurane is recognized as a safe anesthetic, even in patients with long QT syndrome, but it might prolong QTc and/or Tp-e in LQT3 patients and increase the risk of TdP and lethal arrhythmias.
